# The genome sequence of a running crab spider, *Tibellus oblongus* (Walckenaer, 1802) (Araneae: Philodromidae)

**DOI:** 10.12688/wellcomeopenres.25198.1

**Published:** 2025-11-21

**Authors:** Olga Sivell, Sergio Henriques, Natural History Museum Genome Acquisition Lab, Darwin Tree of Life Barcoding Collective, Wellcome Sanger Institute Tree of Life Management, Samples and Laboratory team, Wellcome Sanger Institute Scientific Operations: Sequencing Operations, Wellcome Sanger Institute Tree of Life Core Informatics team, Tree of Life Core Informatics collective, Darwin Tree of Life Consortium

**Affiliations:** 1 Natural History Museum, London, England, UK; 2 Butler University Department of Biological Sciences, Indianapolis, Indiana, USA; 3 Indianapolis Zoological Gardens, Indiana, Indianapolis, USA

**Keywords:** Tibellus oblongus; running crab spider; genome sequence; chromosomal; Araneae

## Abstract

We present a genome assembly from an individual female *Tibellus oblongus* (running crab spider; Arthropoda; Arachnida; Araneae; Philodromidae). The assembly contains two haplotypes with total lengths of 3 189.47 megabases and 3 294.81 megabases. Of haplotype 1 and haplotype 2, 99.03% and 91.65% of the sequence, respectively, are scaffolded into 13 chromosomal pseudomolecules, including X _1_ and X _2_. The mitochondrial genome has also been assembled, with a length of 14.37 kilobases. This assembly was generated as part of the Darwin Tree of Life project, which produces reference genomes for eukaryotic species found in Britain and Ireland.

## Species taxonomy

Eukaryota; Opisthokonta; Metazoa; Eumetazoa; Bilateria; Protostomia; Ecdysozoa; Panarthropoda; Arthropoda; Chelicerata; Arachnida; Araneae; Araneomorphae; Entelegynae; RTA clade; Dionycha; Philodromidae; *Tibellus*; *Tibellus oblongus* (Walckenaer, 1802) (NCBI:txid336685)

## Background

*Tibellus oblongus* (Walckenaer, 1802) is a spider from the family Philodromidae (running crab spiders) and the type species for the genus *Tibellus* Simon, 1875. There is also an accepted subspecies, *Tibellus oblongus maculatus* Caporiacco, 1950 occurring in Italy, however, its validity has been questioned by Ballarin *et al*. (2011) and Pantini & Isaia (2019).

*Tibellus oblongus* occurs in North America, Europe, North Africa, Turkey, Israel, Caucasus, Russia (Europe to Far East), Central Asia, Iran, Mongolia, China, Korea, Japan ( World Spider Catalogue, 2025). In Britain there are two species of *Tibellus*: the common *T. oblongus* and the less common but equally widespread *T. maritimus* (Menge, 1875). They are both straw-coloured, elongated spiders, somewhat resembling *Tetragnatha*, particularly in the way they position their body on vegetation with legs extended in line with grass stems, but with much smaller chelicerae ( Roberts, 1996). Also, unlike *Tetragnatha*, *Tibellus* do not build webs, instead ambushing passing prey.

The two *Tibellus* species are similar in size, with females measuring 8–10 mm and males 7–8 mm. They differ by the colour pattern, with *T. oblongus* having a dark median band on the carapace and abdomen, occasionally with two dark spots at either side at the end of the abdomen, while *T. maritimus* often has dark spots on the sides of carapace and a line of dark spots of both sides of the abdominal median line ( Bee *et al.*, 2017; Bee *et al.*, 2020; Roberts, 1996). However, they can be somewhat variable and examination of genitalia is required for species identification. The epigyne and male pedipalp are distinctive and have been imaged by Locket & Millidge (1951), Brændegaard (1966), Roberts (1996) and Efimik (1999).

The mature individuals can be found from May to July with females occasionally surviving till autumn ( Bee *et al.*, 2017; Bee *et al.*, 2020). Courtship and mating are brief. The egg sac is attached to the top of vegetation such as grasses and guarded by the female which sits on top of it, head down ( Bristowe, 1958; Roberts, 1996). According to Mikulska (1970), this species hibernates in a pre-mature form, reaches maturity in spring, and masses of young emerge in the summer.

*Tibellus oblongus* can be found on grasses, rushes and heather in a wide range of habitats including bogs, sandhills, rough ground, coastal dunes, grassland. This species generally prefers damper conditions than *T. maritimus* and is more common inland ( Bee *et al.*, 2017; Bee *et al.*, 2020; British Arachnological Society, 2025; Roberts, 1996).

**Figure 1.**
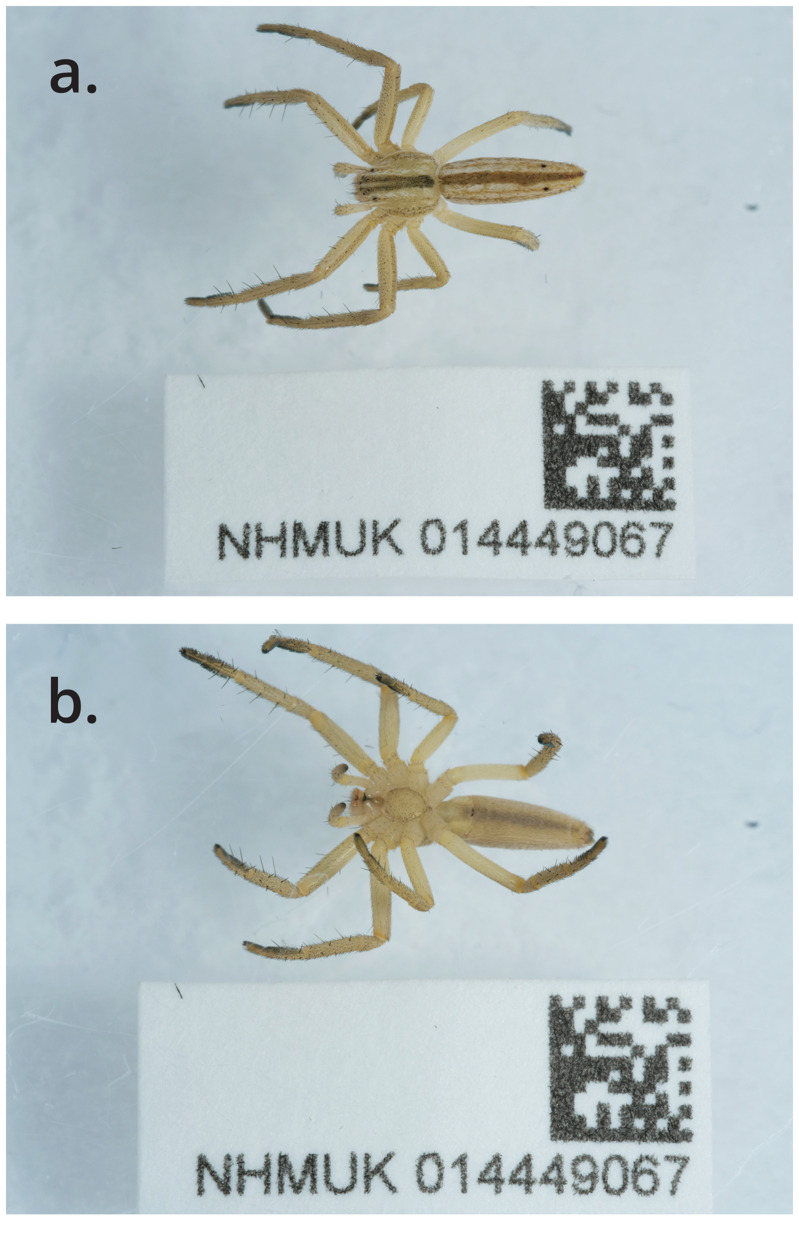
Photographs of the *Tibellus oblongus* (qqTibOblo1) specimen used for genome sequencing.

The high-quality genome of *Tibellus oblongus* was sequenced from a single female (NHMUK014449067; SAMEA9066040) from Wigmore Park, Luton, England ( Figure 1). The genome of *T. oblongus* presented here was sequenced as part of the Darwin Tree of Life Project, a collaborative effort to sequence all named eukaryotic species in the Atlantic Archipelago of Britain and Ireland. This assembly is the first high‑quality genome for the genus *Tibellus* and one of only two genomes available for the family Philodromidae as of October 2025 (NCBI datasets, O’Leary *et al.*, 2024). It will aid research into phylogeny of spiders and taxonomy, biology and ecology of the species.

## Methods

### Sample acquisition and DNA barcoding

The specimen used for genome sequencing was an adult female *Tibellus oblongus* (specimen ID NHMUK014449067, ToLID qqTibOblo1; Figure 1), collected from Wigmore Park, Luton, United Kingdom (latitude 51.884, longitude –0.369) on 2020-10-18. The specimen was collected by Sergio Henriques and Olga Sivell and formally identified by Sergio Henriques. A second specimen was used for Hi-C sequencing (specimen ID NHMUK014537395, ToLID qqTibOblo2). It was collected from University of Exeter Falmouth Campus, Penryn, England, United Kingdom (latitude 50.1712, longitude –5.1233) on 2021-06-30. The specimen was collected by Olga Sivell and identified by Chris Spilling. Sample metadata were collected in line with the Darwin Tree of Life project standards described by Lawniczak *et al*. (2022).

The initial identification was verified by an additional DNA barcoding process according to the framework developed by Twyford *et al.* (2024). A small sample was dissected from the specimen and stored in ethanol, while the remaining parts were shipped on dry ice to the Wellcome Sanger Institute (WSI) (see the protocol). The tissue was lysed, the COI marker region was amplified by PCR, and amplicons were sequenced and compared to the BOLD database, confirming the species identification ( Crowley *et al.*, 2023). Following whole genome sequence generation, the relevant DNA barcode region was also used alongside the initial barcoding data for sample tracking at the WSI ( Twyford *et al.*, 2024). The standard operating procedures for Darwin Tree of Life barcoding are available on protocols.io.

### Nucleic acid extraction

Protocols for high molecular weight (HMW) DNA extraction developed at the Wellcome Sanger Institute (WSI) Tree of Life Core Laboratory are available on protocols.io ( Howard *et al.*, 2025). The qqTibOblo1 sample was weighed and triaged to determine the appropriate extraction protocol. Tissue from the cephalothorax was homogenised by powermashing using a PowerMasher II tissue disruptor. HMW DNA was extracted using the Automated MagAttract v2 protocol. DNA was sheared into an average fragment size of 12–20 kb following the Megaruptor®3 for LI PacBio protocol. Sheared DNA was purified by manual SPRI (solid-phase reversible immobilisation). The concentration of the sheared and purified DNA was assessed using a Nanodrop spectrophotometer and Qubit Fluorometer using the Qubit dsDNA High Sensitivity Assay kit. Fragment size distribution was evaluated by running the sample on the FemtoPulse system.

### PacBio HiFi library preparation and sequencing

Library preparation and sequencing were performed at the WSI Scientific Operations core. Libraries were prepared using the SMRTbell Prep Kit 3.0 (Pacific Biosciences, California, USA), following the manufacturer’s instructions. The kit includes reagents for end repair/A-tailing, adapter ligation, post-ligation SMRTbell bead clean-up, and nuclease treatment. Size selection and clean-up were performed using diluted AMPure PB beads (Pacific Biosciences). DNA concentration was quantified using a Qubit Fluorometer v4.0 (ThermoFisher Scientific) and the Qubit 1X dsDNA HS assay kit. Final library fragment size was assessed with the Agilent Femto Pulse Automated Pulsed Field CE Instrument (Agilent Technologies) using the gDNA 55 kb BAC analysis kit.

The sample was sequenced using the Sequel IIe system (Pacific Biosciences, California, USA). The concentration of the library loaded onto the Sequel IIe was in the range 40–135 pM. The SMRT link software, a PacBio web-based end-to-end workflow manager, was used to set-up and monitor the run, and to perform primary and secondary analysis of the data upon completion.

### Hi-C

***Sample preparation and crosslinking***

The Hi-C sample was prepared from 20–50 mg of frozen tissue of the qqTibOblo2 sample using the Arima-HiC v2 kit (Arima Genomics). Following the manufacturer’s instructions, tissue was fixed and DNA crosslinked using TC buffer to a final formaldehyde concentration of 2%. The tissue was homogenised using the Diagnocine Power Masher-II. Crosslinked DNA was digested with a restriction enzyme master mix, biotinylated, and ligated. Clean-up was performed with SPRISelect beads before library preparation. DNA concentration was measured with the Qubit Fluorometer (Thermo Fisher Scientific) and Qubit HS Assay Kit. The biotinylation percentage was estimated using the Arima-HiC v2 QC beads.

***Hi-C library preparation and sequencing***

Biotinylated DNA constructs were fragmented using a Covaris E220 sonicator and size selected to 400–600 bp using SPRISelect beads. DNA was enriched with Arima-HiC v2 kit Enrichment beads. End repair, A-tailing, and adapter ligation were carried out with the NEBNext Ultra II DNA Library Prep Kit (New England Biolabs), following a modified protocol where library preparation occurs while DNA remains bound to the Enrichment beads. Library amplification was performed using KAPA HiFi HotStart mix and a custom Unique Dual Index (UDI) barcode set (Integrated DNA Technologies). Depending on sample concentration and biotinylation percentage determined at the crosslinking stage, libraries were amplified with 10–16 PCR cycles. Post-PCR clean-up was performed with SPRISelect beads. Libraries were quantified using the AccuClear Ultra High Sensitivity dsDNA Standards Assay Kit (Biotium) and a FLUOstar Omega plate reader (BMG Labtech).

Prior to sequencing, libraries were normalised to 10 ng/μL. Normalised libraries were quantified again and equimolar and/or weighted 2.8 nM pools were created. Pool concentrations were checked using the Agilent 4200 TapeStation (Agilent) with High Sensitivity D500 reagents before sequencing. Sequencing was performed using paired-end 150 bp reads on the Illumina NovaSeq 6000.

### Genome assembly

Prior to assembly of the PacBio HiFi reads, a database of *k*-mer counts ( *k* = 31) was generated from the filtered reads using FastK. GenomeScope2 ( Ranallo-Benavidez *et al.*, 2020) was used to analyse the *k*-mer frequency distributions, providing estimates of genome size, heterozygosity, and repeat content.

The HiFi reads were assembled using Hifiasm in Hi-C phasing mode ( Cheng *et al.*, 2021; Cheng *et al.*, 2022), producing two haplotypes. Hi-C reads ( Rao *et al.*, 2014) were mapped to the primary contigs using bwa-mem2 ( Vasimuddin *et al.*, 2019). Contigs were further scaffolded with Hi-C data in YaHS ( Zhou *et al.*, 2023), using the --break option for handling potential misassemblies. The scaffolded assemblies were evaluated using Gfastats ( Formenti *et al.*, 2022), BUSCO ( Manni *et al.*, 2021) and MERQURY.FK ( Rhie *et al.*, 2020). The organelle genomes were assembled using MitoHiFi ( Uliano-Silva *et al.*, 2023).

### Assembly curation

The assembly was decontaminated using the Assembly Screen for Cobionts and Contaminants ( ASCC) pipeline. TreeVal was used to generate the flat files and maps for use in curation. Manual curation was conducted primarily in PretextView and HiGlass ( Kerpedjiev *et al.*, 2018). Scaffolds were visually inspected and corrected as described by Howe *et al*. (2021). Manual corrections included 96 breaks and 233 joins. This reduced the scaffold count by 9.3%, reduced the scaffold N50 by 4.7%, and reduced the total assembly length by 3.9%. The curation process is documented at https://gitlab.com/wtsi-grit/rapid-curation. PretextSnapshot was used to generate a Hi-C contact map of the final assembly.

### Assembly quality assessment

The Merqury.FK tool ( Rhie *et al.*, 2020) was run in a Singularity container ( Kurtzer *et al.*, 2017) to evaluate *k*-mer completeness and assembly quality for both haplotypes using the *k*-mer databases ( *k* = 31) computed prior to genome assembly. The analysis outputs included assembly QV scores and completeness statistics.

The genome was analysed using the BlobToolKit pipeline, a Nextflow implementation of the earlier Snakemake version ( Challis *et al.*, 2020). The pipeline aligns PacBio reads using minimap2 ( Li, 2018) and SAMtools ( Danecek *et al.*, 2021) to generate coverage tracks. It runs BUSCO ( Manni *et al.*, 2021) using lineages identified from the NCBI Taxonomy ( Schoch *et al.*, 2020). For the three domain-level lineages, BUSCO genes are aligned to the UniProt Reference Proteomes database ( Bateman *et al.*, 2023) using DIAMOND blastp ( Buchfink *et al.*, 2021). The genome is divided into chunks based on the density of BUSCO genes from the closest taxonomic lineage, and each chunk is aligned to the UniProt Reference Proteomes database with DIAMOND blastx. Sequences without hits are chunked using seqtk and aligned to the NT database with blastn ( Altschul *et al.*, 1990). The BlobToolKit suite consolidates all outputs into a blobdir for visualisation. The BlobToolKit pipeline was developed using nf-core tooling ( Ewels *et al.*, 2020) and MultiQC ( Ewels *et al.*, 2016), with containerisation through Docker ( Merkel, 2014) and Singularity ( Kurtzer *et al.*, 2017).

## Genome sequence report

### Sequence data

**Figure 2.**
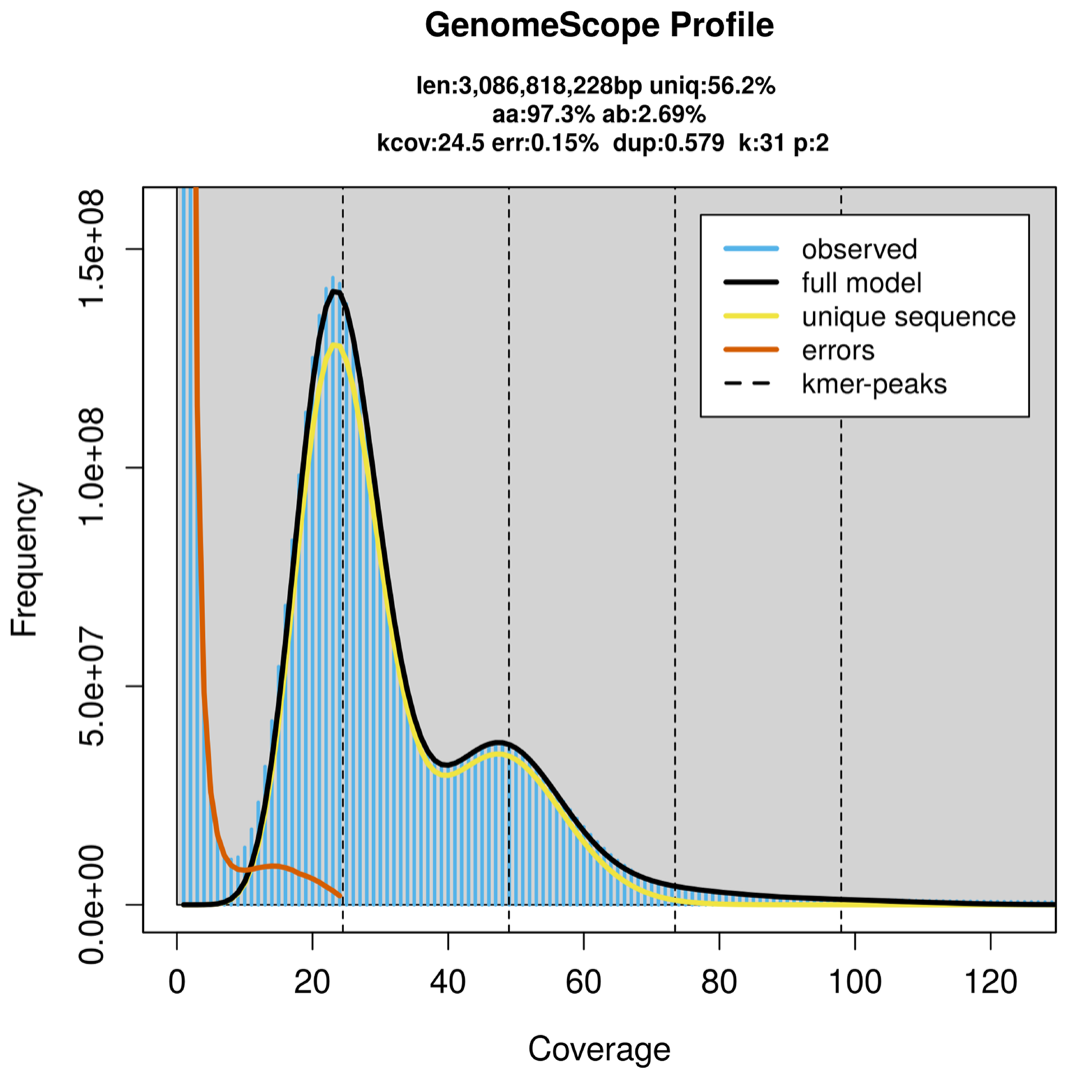
Frequency distribution of *k*-mers generated using GenomeScope2. The plot shows observed and modelled *k*-mer spectra, providing estimates of genome size, heterozygosity, and repeat content based on unassembled sequencing reads.

PacBio sequencing of the *Tibellus oblongus* specimen generated 140.62 Gb (gigabases) from 14.44 million reads, which were used to assemble the genome. GenomeScope2.0 analysis estimated the haploid genome size at 3 086.82 Mb, with a heterozygosity of 2.69% and repeat content of 43.98% ( Figure 2). These estimates guided expectations for the assembly. Based on the estimated genome size, the sequencing data provided approximately 49× coverage. Hi-C sequencing produced 537.04 Gb from 3 556.56 million reads, which were used to scaffold the assembly. Table 1 summarises the specimen and sequencing details.

**Table 1.** Specimen and sequencing data for BioProject PRJEB73938.

Platform	PacBio HiFi	Hi-C
**ToLID**	qqTibOblo1	qqTibOblo2
**Specimen ID**	NHMUK014449067	NHMUK014537395
**BioSample (source individual)**	SAMEA9066040	SAMEA11025078
**BioSample (tissue)**	SAMEA9066131	SAMEA11025334
**Tissue**	cephalothorax	whole organism tissue
**Instrument**	Sequel IIe	Illumina NovaSeq 6000
**Run accessions**	ERR12760822; ERR12760823; ERR12760826; ERR12760825; ERR12760824	ERR12765199
**Read count total**	14.44 million	3 556.56 million
**Base count total**	140.62 Gb	537.04 Gb

### Assembly statistics

The genome was assembled into two haplotypes using Hi-C phasing. Haplotype 1 was curated to chromosome level, while haplotype 2 was assembled to scaffold level. The final assembly has a total length of 3 189.47 Mb in 242 scaffolds, with 323 gaps, and a scaffold N50 of 239.92 Mb ( Table 2).

**Table 2.** Genome assembly statistics.

**Assembly name**	qqTibOblo1.hap1.1	qqTibOblo1.hap2.1
**Assembly accession**	GCA_965643835.1	GCA_965643845.1
**Assembly level**	chromosome	chromosome
**Span (Mb)**	3 189.47	3 294.81
**Number of chromosomes**	13	13
**Number of contigs**	565	10 976
**Contig N50**	48.52 Mb	3.02 Mb
**Number of scaffolds**	242	8 639
**Scaffold N50**	239.92 Mb	232.5 Mb
**Longest scaffold length (Mb)**	350.93	346.13
**Sex chromosomes**	X _1_ and X _2_	X _1_ and X _2_
**Organelles**	Mitochondrion: 14.37 kb	-

**Figure 3.**
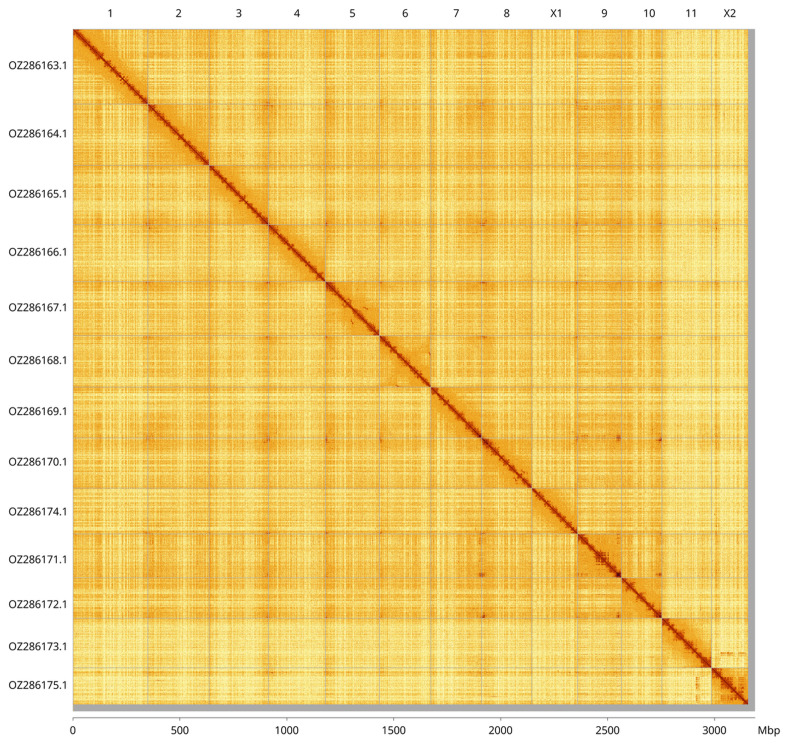
Hi-C contact map of the *Tibellus oblongus* genome assembly. Assembled chromosomes are shown in order of size and labelled along the axes, with a megabase scale shown below. The plot was generated using PretextSnapshot.

Most of the haplotype 1 assembly sequence (99.03%) was assigned to 13 chromosomal-level scaffolds, representing 11 autosomes and the X1 and X2 sex chromosomes. These chromosome-level scaffolds, confirmed by Hi-C data, are named according to size ( Figure 3; Table 3). Chromosomes X _1_ and X2 were identified by copy number in the diploid assembly and BUSCO synteny with the chromosomes of *Dolomedes plantarius* (GCA_907164885.2). During curation we observed a haplotypic inversion in the region on chromosome 5 (85.0–111.6, 124.1–186.9 Mbp), chromosome 6 (83.2–234.0 Mbp) and chromosome 8 (156.9–189.1 Mbp). We also noted that the regions on chromosome 11 (0–23.5 Mbp) and chromosome X _2_ (16.1–31.4 Mbp) were collapsed in haplotype 2

**Table 3.** Chromosomal pseudomolecules in both haplotypes of the genome assembly of *Tibellus oblongus*, qqTibOblo1.

Haplotype 1				Haplotype 2			
INSDC accession	Name	Length (Mb)	GC%	INSDC accession	Name	Length (Mb)	GC%
OZ286163.1	1	350.93	31.50	OZ286184.1	1	346.13	31.50
OZ286164.1	2	286.67	32	OZ286185.1	2	278.66	32
OZ286165.1	3	276.90	31.50	OZ286186.1	3	268.44	31.50
OZ286166.1	4	266.72	31.50	OZ286187.1	4	258.82	31.50
OZ286167.1	5	252.80	31.50	OZ286188.1	5	241.41	31.50
OZ286168.1	6	239.92	31.50	OZ286189.1	6	233.22	31.50
OZ286169.1	7	237.86	31.50	OZ286177.1	7	232.50	31.50
OZ286170.1	8	234	31.50	OZ286178.1	8	225.18	31.50
OZ286171.1	9	214.89	31	OZ286179.1	9	206.07	31
OZ286172.1	10	204.41	32.50	OZ286180.1	10	191.35	32.50
OZ286173.1	11	189.14	32	OZ286181.1	11	161.17	31.50
OZ286174.1	X _1_	232.08	31	OZ286182.1	X _1_	224.38	31
OZ286175.1	X _2_	172.14	31.50	OZ286183.1	X _2_	152.36	31.50

The mitochondrial genome was also assembled (length 14.37 kb, OZ286176.1). This sequence is included as a contig in the multifasta file of the genome submission and as a standalone record.

**Figure 4.**
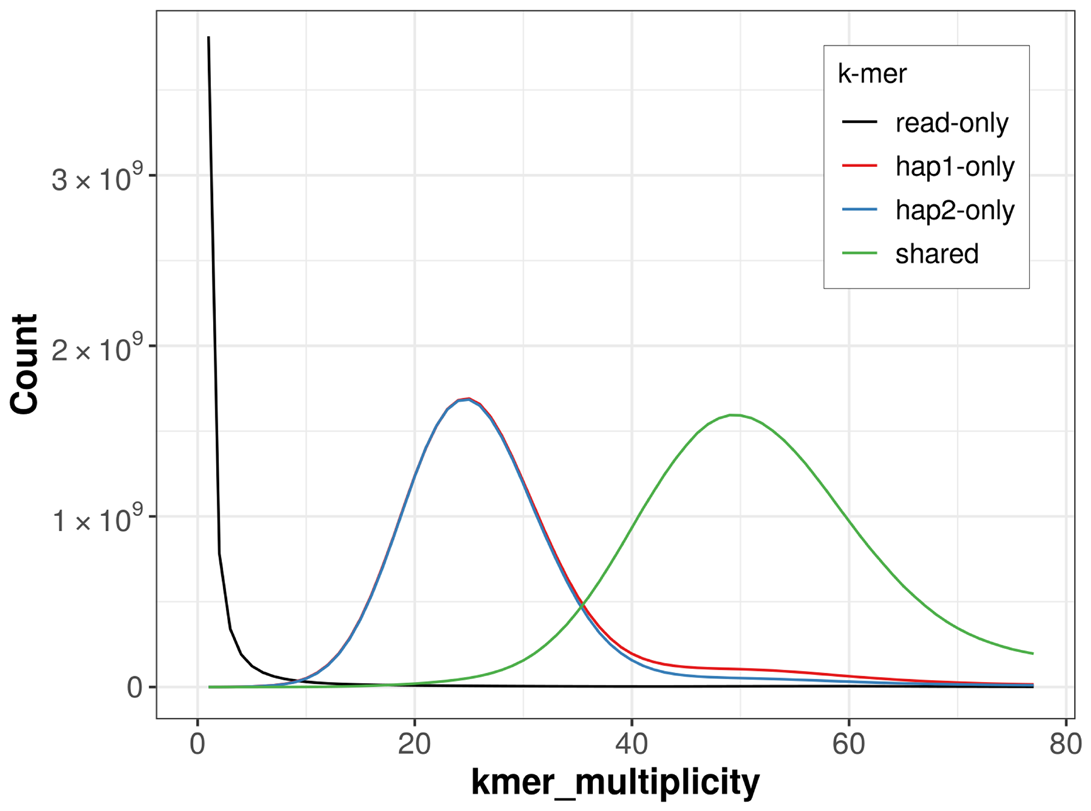
Evaluation of *k*-mer completeness using MerquryFK. This plot illustrates the recovery of *k*-mers from the original read data in the final assemblies. The horizontal axis represents *k*-mer multiplicity, and the vertical axis shows the number of *k*-mers. The black curve represents *k*-mers that appear in the reads but are not assembled. The green curve corresponds to *k*-mers shared by both haplotypes, and the red and blue curves show *k*-mers found only in one of the haplotypes.

For haplotype 1, the estimated QV is 59.5, and for haplotype 2, 58.9. When the two haplotypes are combined, the assembly achieves an estimated QV of 59.2. The *k*-mer completeness is 64.87% for haplotype 1, 63.74% for haplotype 2, and 99.24% for the combined haplotypes ( Figure 4).

**Figure 5.**
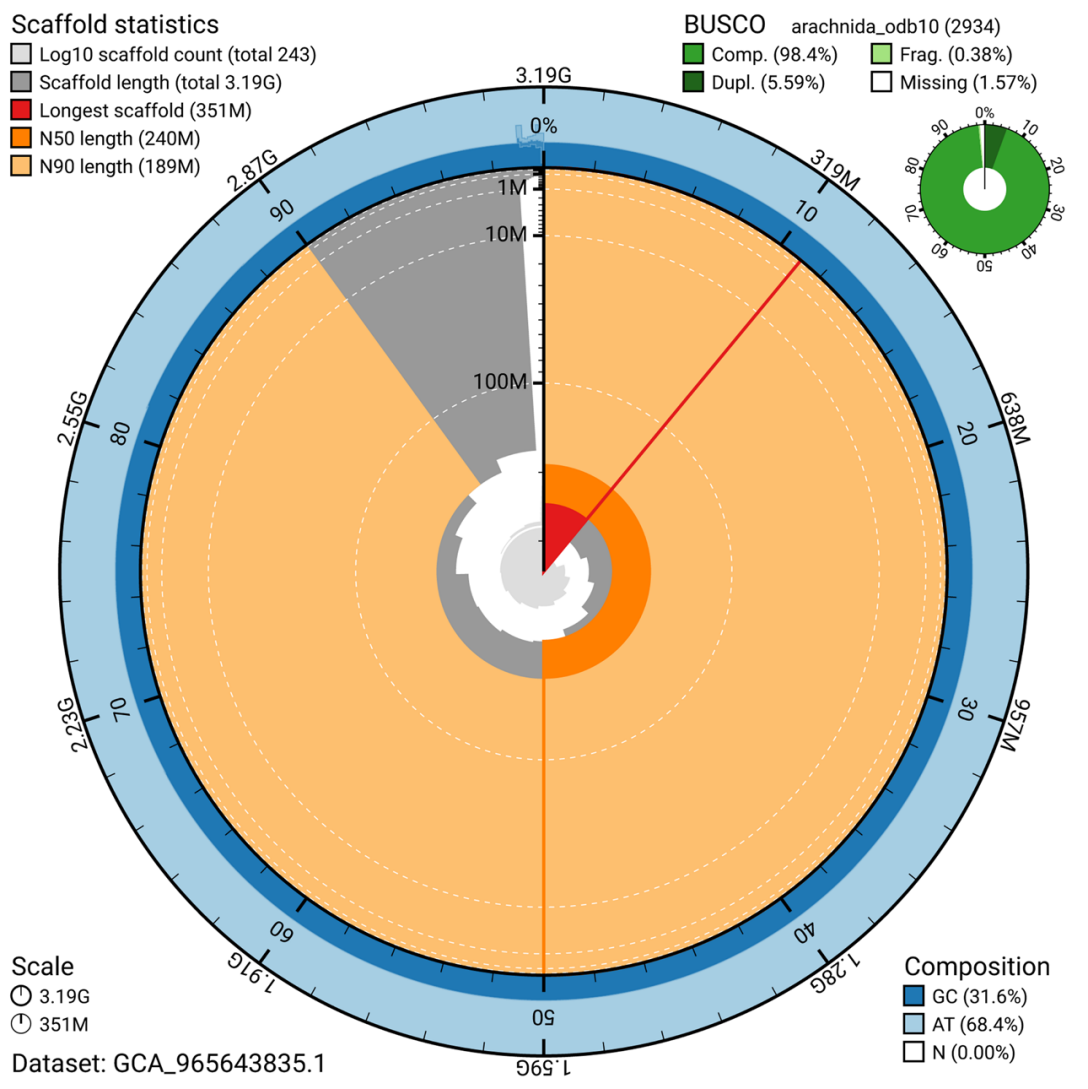
Assembly metrics for qqTibOblo1.hap1.1. The BlobToolKit snail plot provides an overview of assembly metrics and BUSCO gene completeness. The circumference represents the length of the whole genome sequence, and the main plot is divided into 1 000 bins around the circumference. The outermost blue tracks display the distribution of GC, AT, and N percentages across the bins. Scaffolds are arranged clockwise from longest to shortest and are depicted in dark grey. The longest scaffold is indicated by the red arc, and the deeper orange and pale orange arcs represent the N50 and N90 lengths. A light grey spiral at the centre shows the cumulative scaffold count on a logarithmic scale. A summary of complete, fragmented, duplicated, and missing BUSCO genes in the set is presented at the top right. An interactive version of this figure can be accessed on the BlobToolKit viewer.

**Figure 6.**
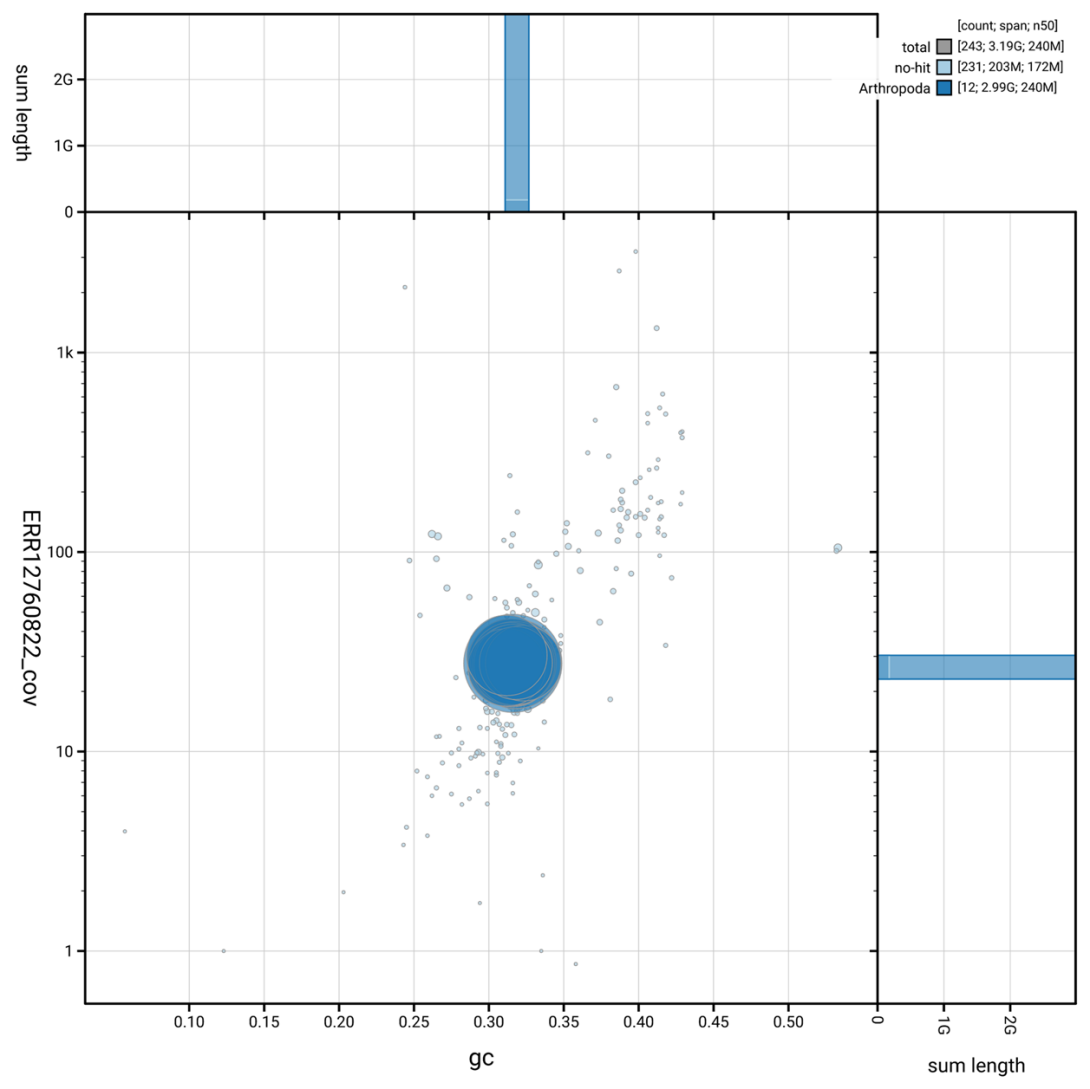
BlobToolKit GC-coverage plot for qqTibOblo1.hap1.1. Blob plot showing sequence coverage (vertical axis) and GC content (horizontal axis). The circles represent scaffolds, with the size proportional to scaffold length and the colour representing phylum membership. The histograms along the axes display the total length of sequences distributed across different levels of coverage and GC content. An interactive version of this figure is available on the BlobToolKit viewer.

BUSCO analysis using the arachnida_odb10 reference set ( *n* = 2 934) identified 98.4% of the expected gene set (single = 92.8%, duplicated = 5.6%) for haplotype 1. The snail plot in Figure 5 summarises the scaffold length distribution and other assembly statistics for haplotype 1. The blob plot in Figure 6 shows the distribution of scaffolds by GC proportion and coverage for haplotype 1.

Table 4 lists the assembly metric benchmarks adapted from Rhie *et al.* (2021) and the Earth BioGenome Project Report on Assembly Standards September 2024. The EBP metric, calculated for the haplotype 1, is **7.C.Q59**, meeting the recommended reference standard.

**Table 4.** Earth Biogenome Project summary metrics for the *Tibellus oblongus* assembly.

Measure	Value	Benchmark
EBP summary (haplotype 1)	7.C.Q59	6.C.Q40
Contig N50 length	48.52 Mb	≥ 1 Mb
Scaffold N50 length	239.92 Mb	= chromosome N50
Consensus quality (QV)	Haplotype 1: 59.5; haplotype 2: 58.9; combined: 59.2	≥ 40
*k*-mer completeness	Haplotype 1: 64.87%; Haplotype 2: 63.74%; combined: 99.24%	≥ 95%
BUSCO	C:98.4% [S:92.8%; D:5.6%]; F:0.4%; M:1.2%; n:2 934	S > 90%; D < 5%
Percentage of assembly assigned to chromosomes	99.03%	≥ 90%

### Wellcome Sanger Institute – Legal and Governance

The materials that have contributed to this genome note have been supplied by a Darwin Tree of Life Partner. The submission of materials by a Darwin Tree of Life Partner is subject to the **‘Darwin Tree of Life Project Sampling Code of Practice’**, which can be found in full on the Darwin Tree of Life website. By agreeing with and signing up to the Sampling Code of Practice, the Darwin Tree of Life Partner agrees they will meet the legal and ethical requirements and standards set out within this document in respect of all samples acquired for, and supplied to, the Darwin Tree of Life Project. Further, the Wellcome Sanger Institute employs a process whereby due diligence is carried out proportionate to the nature of the materials themselves, and the circumstances under which they have been/are to be collected and provided for use. The purpose of this is to address and mitigate any potential legal and/or ethical implications of receipt and use of the materials as part of the research project, and to ensure that in doing so we align with best practice wherever possible. The overarching areas of consideration are:

- Ethical review of provenance and sourcing of the material
- Legality of collection, transfer and use (national and international)

Each transfer of samples is further undertaken according to a Research Collaboration Agreement or Material Transfer Agreement entered into by the Darwin Tree of Life Partner, Genome Research Limited (operating as the Wellcome Sanger Institute), and in some circumstances, other Darwin Tree of Life collaborators.

## Data Availability

European Nucleotide Archive: Tibellus oblongus. Accession number PRJEB73938. The genome sequence is released openly for reuse. The *Tibellus oblongus* genome sequencing initiative is part of the Darwin Tree of Life Project (PRJEB40665) and the Sanger Institute Tree of Life Programme (PRJEB43745). All raw sequence data and the assembly have been deposited in INSDC databases. The genome will be annotated using available RNA-Seq data and presented through the Ensembl pipeline at the European Bioinformatics Institute. Raw data and assembly accession identifiers are reported in Table 1 and Table 2. Production code used in genome assembly at the WSI Tree of Life is available at https://github.com/sanger-tol. Table 5 lists software versions used in this study.

## Author information

Contributors are listed at the following links:

- Members of the Natural History Museum Genome Acquisition Lab
- Members of the Darwin Tree of Life Barcoding collective
- Members of the Wellcome Sanger Institute Tree of Life Management, Samples and Laboratory team
- Members of Wellcome Sanger Institute Scientific Operations – Sequencing Operations
- Members of the Wellcome Sanger Institute Tree of Life Core Informatics team
- Members of the Tree of Life Core Informatics collective
- Members of the Darwin Tree of Life Consortium

## References

[ref-1] Altschul SF Gish W Miller W : Basic Local Alignment Search Tool. *J Mol Biol.* 1990;215(3):403–410. 10.1016/S0022-2836(05)80360-2 2231712

[ref-2] Ballarin F Pantini P Hansen H : Catalogo ragionato dei ragni (Arachnida, Araneae) del Veneto. *Memorie del Museo Civico di Storia Naturale di Verona, 2. serie, sezione biologica.* 2011;21. Reference Source

[ref-3] Bateman A Martin MJ Orchard S : UniProt: the Universal Protein Knowledgebase in 2023. *Nucleic Acids Res.* 2023;51(D1):D523–D531. 10.1093/nar/gkac1052 36408920 PMC9825514

[ref-4] Bee L Oxford G Smith H : Britain’s spiders: a field guide.WILDGuides, Princeton University Press,2017. Reference Source

[ref-5] Bee L Oxford G Smith H : Britain’s spiders: a field guide – fully revised and updated second edition.WILDGuides, Princeton University Press,2020. Reference Source

[ref-6] Brændegaard J : Edderkopper: eller spindlere. 1966. Reference Source

[ref-7] Bristowe WS : The world of spiders.London: Collins,1958. Reference Source

[ref-8] British Arachnological Society: Summary for *Tibellus oblongus* (Araneae) in the spider and harvestman recording scheme. 2025. Reference Source

[ref-9] Buchfink B Reuter K Drost HG : Sensitive protein alignments at Tree-of-Life scale using DIAMOND. *Nat Methods.* 2021;18(4):366–368. 10.1038/s41592-021-01101-x 33828273 PMC8026399

[ref-10] Challis R Richards E Rajan J : BlobToolKit – interactive quality assessment of genome assemblies. *G3 (Bethesda).* 2020;10(4):1361–1374. 10.1534/g3.119.400908 32071071 PMC7144090

[ref-11] Cheng H Concepcion GT Feng X : Haplotype-resolved *de novo* assembly using phased assembly graphs with hifiasm. *Nat Methods.* 2021;18(2):170–175. 10.1038/s41592-020-01056-5 33526886 PMC7961889

[ref-12] Cheng H Jarvis ED Fedrigo O : Haplotype-resolved assembly of diploid genomes without parental data. *Nat Biotechnol.* 2022;40(9):1332–35. 10.1038/s41587-022-01261-x 35332338 PMC9464699

[ref-13] Crowley L Allen H Barnes I : A sampling strategy for genome sequencing the British terrestrial arthropod fauna [version 1; peer review: 2 approved]. *Wellcome Open Res.* 2023;8:123. 10.12688/wellcomeopenres.18925.1 37408610 PMC10318377

[ref-14] Danecek P Bonfield JK Liddle J : Twelve years of SAMtools and BCFtools. *GigaScience.* 2021;10(2): giab008. 10.1093/gigascience/giab008 33590861 PMC7931819

[ref-15] Efimik VE : A review of the spider genus *Tibellus* (Simon, 1875) of the East Palaearctic (Aranei: Philodromidae). *Arthropoda Selecta.* 1999;8:103–24.

[ref-16] Ewels P Magnusson M Lundin S : MultiQC: summarize analysis results for multiple tools and samples in a single report. *Bioinformatics.* 2016;32(19):3047–3048. 10.1093/bioinformatics/btw354 27312411 PMC5039924

[ref-17] Ewels PA Peltzer A Fillinger S : The nf-core framework for community-curated bioinformatics pipelines. *Nat Biotechnol.* 2020;38(3):276–278. 10.1038/s41587-020-0439-x 32055031

[ref-18] Formenti G Abueg L Brajuka A : Gfastats: conversion, evaluation and manipulation of genome sequences using assembly graphs. *Bioinformatics.* 2022;38(17):4214–4216. 10.1093/bioinformatics/btac460 35799367 PMC9438950

[ref-19] Howard C Denton A Jackson B : On the path to reference genomes for all biodiversity: lessons learned and laboratory protocols created in the Sanger Tree of Life core laboratory over the first 2000 species. *bioRxiv.* 2025. 10.1101/2025.04.11.648334 PMC12548527 41129326

[ref-20] Howe K Chow W Collins J : Significantly improving the quality of genome assemblies through curation. *GigaScience.* 2021;10(1): giaa153. 10.1093/gigascience/giaa153 33420778 PMC7794651

[ref-21] Kerpedjiev P Abdennur N Lekschas F : HiGlass: web-based visual exploration and analysis of genome interaction maps. *Genome Biol.* 2018;19(1): 125. 10.1186/s13059-018-1486-1 30143029 PMC6109259

[ref-22] Kurtzer GM Sochat V Bauer MW : Singularity: scientific containers for mobility of compute. *PLoS One.* 2017;12(5): e0177459. 10.1371/journal.pone.0177459 28494014 PMC5426675

[ref-23] Lawniczak MKN Davey RP Rajan J : Specimen and sample metadata standards for biodiversity genomics: a proposal from the Darwin Tree of Life Project [version 1; peer review: 2 approved with reservations]. *Wellcome Open Res.* 2022;7:187. 10.12688/wellcomeopenres.17605.1

[ref-24] Li H : Minimap2: pairwise alignment for nucleotide sequences. *Bioinformatics.* 2018;34(18):3094–3100. 10.1093/bioinformatics/bty191 29750242 PMC6137996

[ref-25] Locket GH Millidge AF : British spiders. Vol. I. London: Ray Society, 1951;1. Reference Source

[ref-26] Manni M Berkeley MR Seppey M : BUSCO update: novel and streamlined workflows along with broader and deeper phylogenetic coverage for scoring of eukaryotic, prokaryotic, and viral genomes. *Mol Biol Evol.* 2021;38(10):4647–4654. 10.1093/molbev/msab199 34320186 PMC8476166

[ref-27] Merkel D : Docker: lightweight Linux containers for consistent development and deployment. *Linux J.* 2014;2014(239): 2. Reference Source

[ref-28] Mikulska I : Some observations on the biology of the spider *Tibellus oblongus* (Walckenaer) (Araneae). *Bull Br Arachnol Soc.* 1970;1:113–16. Reference Source

[ref-29] O’Leary NA Cox E Holmes JB : Exploring and retrieving sequence and metadata for species across the Tree of Life with NCBI datasets. *Sci Data.* 2024;11(1): 732. 10.1038/s41597-024-03571-y 38969627 PMC11226681

[ref-30] Pantini P Isaia M : Araneae.it: the online catalog of Italian spiders, with addenda on other arachnid orders occurring in Italy (Arachnida: Araneae, Opiliones, Palpigradi, Pseudoscorpionida, Scorpiones, Solifugae). *Fragmenta Entomologica.* 2019;51(2):127–52. 10.13133/2284-4880/374

[ref-31] Ranallo-Benavidez TR Jaron KS Schatz MC : GenomeScope 2.0 and Smudgeplot for reference-free profiling of polyploid genomes. *Nat Commun.* 2020;11(1): 1432. 10.1038/s41467-020-14998-3 32188846 PMC7080791

[ref-32] Rao SSP Huntley MH Durand NC : A 3D map of the human genome at kilobase resolution reveals principles of chromatin looping. *Cell.* 2014;159(7):1665–1680. 10.1016/j.cell.2014.11.021 25497547 PMC5635824

[ref-33] Rhie A McCarthy SA Fedrigo O : Towards complete and error-free genome assemblies of all vertebrate species. *Nature.* 2021;592(7856):737–746. 10.1038/s41586-021-03451-0 33911273 PMC8081667

[ref-34] Rhie A Walenz BP Koren S : Merqury: reference-free quality, completeness, and phasing assessment for genome assemblies. *Genome Biol.* 2020;21(1): 245. 10.1186/s13059-020-02134-9 32928274 PMC7488777

[ref-35] Roberts MJ : Collins field guide: spiders of Britain & Northern Europe.London: HarperCollins,1996.

[ref-36] Schoch CL Ciufo S Domrachev M : NCBI Taxonomy: a comprehensive update on curation, resources and tools. *Database (Oxford).* 2020;2020: baaa062. 10.1093/database/baaa062 32761142 PMC7408187

[ref-37] Twyford AD Beasley J Barnes I : A DNA barcoding framework for taxonomic verification in the Darwin Tree of Life Project [version 1; peer review: 2 approved]. *Wellcome Open Res.* 2024;9:339. 10.12688/wellcomeopenres.21143.1 39386966 PMC11462125

[ref-38] Uliano-Silva M Ferreira JGRN Krasheninnikova K : MitoHiFi: a python pipeline for mitochondrial genome assembly from PacBio high fidelity reads. *BMC Bioinformatics.* 2023;24(1): 288. 10.1186/s12859-023-05385-y 37464285 PMC10354987

[ref-39] Vasimuddin M Misra S Li H : Efficient architecture-aware acceleration of BWA-MEM for multicore systems.In: *2019 IEEE International Parallel and Distributed Processing Symposium (IPDPS).*IEEE,2019;314–324. 10.1109/IPDPS.2019.00041

[ref-40] World Spider Catalogue: World spider catalogue: Tibellus oblongus. 2025. Reference Source

[ref-41] Zhou C McCarthy SA Durbin R : YaHS: Yet another Hi-C Scaffolding tool. *Bioinformatics.* 2023;39(1): btac808. 10.1093/bioinformatics/btac808 36525368 PMC9848053

