# Supplementary material for: The genome sequence of a running crab spider, Tibellus oblongus (Walckenaer, 1802) (Araneae: Philodromidae)
Source: Wellcome Open Res. 2025 Nov 21;10:651. [Version 1] doi: 10.12688/wellcomeopenres.25198.1 (PMC13109704; doi:10.12688/wellcomeopenres.25198.1)
Supplement: Peer review history

# Reviewer report: Reviewer response for version 1

**DOI:** 10.21956/wellcomeopenres.27774.r153543

**Published:** 2026-04-24

**Authors:** Yannis Schöneberg

**Affiliations:** 1 Trier University, trier, Germany

The authors provide a high quality, phased genome for the running crab spider *Tibellus oblongus *as a data resource. One of the haplotypes was assembled to chromsome level, for the other, the authors reached a highly contiguous scaffold level. Methods are meticulously documented. The genome will represent a highly valuable resource for future research. 

 Therefore, I would like to congratulate the authors to their carefully prepared manuscript. I only have a single, minor comment.

 Detailed comments:

 1: In Table 3 chromosomal pseudomolecules between haplotypes are compared. However, before it was mentioned that haplotype 2 did not reach chromosome level. I think it would be good to briefly explain how chromosomal pseudomolecules for haplotype 2 were chosen.

Are sufficient details of methods and materials provided to allow replication by others?

Yes

Is the rationale for creating the dataset(s) clearly described?

Yes

Are the datasets clearly presented in a useable and accessible format?

Yes

Are the protocols appropriate and is the work technically sound?

Yes

Reviewer Expertise:

genomics of spiders, comparative genomics

I confirm that I have read this submission and believe that I have an appropriate level of expertise to confirm that it is of an acceptable scientific standard.

# Reviewer report: Reviewer response for version 1

**DOI:** 10.21956/wellcomeopenres.27774.r148747

**Published:** 2026-03-04

**Authors:** Marc Domènech

**Affiliations:** 1 Universitat de Barcelona, Barcelona, Spain

This article provides the first reference genome for the spider *Tibellus oblongus*, scaffolded into 13 pseudochromosomes. The introduction comments on its distribution, taxonomy and biology, while the methods section clearly outlines the protocol from sample acquisition to genome assembly. The species identification was confirmed by means of DNA Barcoding, which is positive. This genome is a useful resource that can aid studies on the phylogeny, biology and ecology of this group.

 I have only two minor comments regarding the introduction:

 -The body size is said to be 8-10mm for females and 7-8mm males. Where do these measurements come from? According to both araneae.nmbe.ch and the World Spider Trait database the ranges seem to be quite larger (6.5-11-5 in females, 5-7.5 in males).

 -Adults are mentioned to be present May to July, ocasionally to autumn. Does this information refer to the UK specifically? Some adults seem to be found earlier too, at least in some parts of its range (https://araneae.nmbe.ch/data/901)

Are sufficient details of methods and materials provided to allow replication by others?

Yes

Is the rationale for creating the dataset(s) clearly described?

Yes

Are the datasets clearly presented in a useable and accessible format?

Yes

Are the protocols appropriate and is the work technically sound?

Yes

Reviewer Expertise:

Arachnology, Systematics, Taxonomy

I confirm that I have read this submission and believe that I have an appropriate level of expertise to confirm that it is of an acceptable scientific standard.
